# Clofazimine pharmacokinetics in patients with TB: dosing implications

**DOI:** 10.1093/jac/dkaa310

**Published:** 2020-08-03

**Authors:** Mahmoud Tareq Abdelwahab, Sean Wasserman, James C M Brust, Neel R Gandhi, Graeme Meintjes, Daniel Everitt, Andreas Diacon, Rodney Dawson, Lubbe Wiesner, Elin M Svensson, Gary Maartens, Paolo Denti

**Affiliations:** d1 Division of Clinical Pharmacology, Department of Medicine, University of Cape Town, Cape Town, South Africa; d2 Division of Infectious Diseases and HIV Medicine, Department of Medicine, University of Cape Town, Cape Town, South Africa; d3 Wellcome Centre for Infectious Diseases Research in Africa, Institute of Infectious Disease and Molecular Medicine, University of Cape Town, Cape Town, South Africa; d4 Divisions of General Internal Medicine and Infectious Diseases, Albert Einstein College of Medicine, New York, NY, USA; d5 Departments of Epidemiology and Global Health, Rollins School of Public Health, Emory University, Atlanta, GA, USA; d6 Department of Medicine (Infectious Diseases), Emory School of Medicine, Emory University, Atlanta, GA, USA; d7 Global Alliance for TB Drug Development, New York, NY, USA; d8 Task Applied Science, Bellville, and Department of Medicine, Stellenbosch University, Tygerberg, Cape Town, South Africa; d9 University of Cape Town Lung Institute and Division of Pulmonology, Department of Medicine, University of Cape Town, Cape Town, South Africa; d10 Department of Pharmacy, Radboud Institute of Health Sciences, Radboud University Medical Center, Nijmegen, The Netherlands; d11 Department of Pharmaceutical Biosciences, Uppsala University, Uppsala, Sweden

## Abstract

**Background:**

Clofazimine is in widespread use as a key component of drug-resistant TB regimens, but the recommended dose is not evidence based. Pharmacokinetic data from relevant patient populations are needed to inform dose optimization.

**Objectives:**

To determine clofazimine exposure, evaluate covariate effects on variability, and simulate exposures for different dosing strategies in South African TB patients.

**Patients and methods:**

Clinical and pharmacokinetic data were obtained from participants with pulmonary TB enrolled in two studies with intensive and sparse sampling for up to 6 months. Plasma concentrations were measured by LC-MS/MS and interpreted with non-linear mixed-effects modelling. Body size descriptors and other potential covariates were tested on pharmacokinetic parameters. We simulated different dosing regimens to safely shorten time to average daily concentration above a putative target concentration of 0.25 mg/L.

**Results:**

We analysed 1570 clofazimine concentrations from 139 participants; 79 (57%) had drug-resistant TB and 54 (39%) were HIV infected. Clofazimine pharmacokinetics were well characterized by a three-compartment model. Clearance was 11.5 L/h and peripheral volume 10 500 L for a typical participant. Lower plasma exposures were observed in women during the first few months of treatment, explained by higher body fat fraction. Model-based simulations estimated that a loading dose of 200 mg daily for 2 weeks would achieve average daily concentrations above a target efficacy concentration 37 days earlier in a typical TB participant.

**Conclusions:**

Clofazimine was widely distributed with a long elimination half-life. Disposition was strongly influenced by body fat content, with potential dosing implications for women with TB.

## Introduction

Drug-resistant TB remains a major obstacle to achieving ‘End TB’ targets.^1^ A key driver of the drug-resistant TB epidemic has been a lack of effective therapy, leading to low cure rates, amplification of resistance and ongoing transmission. New therapeutic options with improved efficacy and tolerability have recently become available, including the new anti-TB agents bedaquiline and delamanid, and the repurposed drugs linezolid and clofazimine. As a result, there has been a shift in treatment guidelines towards shorter injection-free regimens.^2^

Clofazimine is recommended by the WHO and the American Thoracic Society for patients with rifampicin-resistant TB^3^^,^^4^ and also has a potential role in treatment-shortening regimens for drug-susceptible TB.^5^ First discovered in 1957, clofazimine has been used almost exclusively in combination therapy for leprosy. There have been no studies evaluating dose–exposure relationships in patients with drug-resistant TB, and the optimal dose for this condition is unknown. Clofazimine is highly protein bound^6^ and undergoes duration-dependent accumulation in fat, tissue macrophages and reticuloendothelial organs, resulting in an extremely long terminal half-life,^7^ with implications for risk of adverse effects and emergence of resistance. Furthermore, because clofazimine may be a substrate of cytochrome P450 (CYP) and the P-glycoprotein transporter,^8^^,^^9^ there are potential pharmacokinetic (PK) drug–drug interactions, including with antiretrovirals and other anti-TB agents. Though such research has been undertaken, it is unknown how well murine models of clofazimine dosing predict human PK.^10^ As a consequence of this limitation, plus the lack of PK data from TB patients, pharmacodynamic (PD) targets for efficacy and toxicity for clofazimine in TB treatment have not been established.

A model-based approach that can account for the unusual PK characteristics of clofazimine and predict individual exposures is required to estimate dose–response relationships and inform dose optimization in TB treatment.^11^ Using data from two cohorts of South African patients with TB, we developed a model to describe the population PK of clofazimine, evaluate covariate effects on PK variability and simulate exposures for optimized dosing strategies.

## Patients and methods

### Study design and population

Clinical and drug concentration data were obtained from a prospective observational cohort study (PROBeX) of adults treated with bedaquiline for pulmonary XDR and pre-XDR TB recruited from three TB hospitals in South Africa. Most participants were treated with regimens that also included clofazimine 100 mg daily, as per local standard of care. Detailed clinical and laboratory data were collected at monthly study visits over 6 months. Sparse PK sampling was performed at roughly 1, 2 and 6 months after starting clofazimine at a single pre-dose timepoint after self-reported dosing. A subgroup of consecutive participants at a single site consented to intensive sampling (pre-dose and at 1, 2, 3, 4, 5, 6, 8 and 24 h after an observed dose and a standard meal of peanut butter on brown bread) at the Month 2 visit. Clofazimine plasma concentrations were measured at the Division of Clinical Pharmacology at the University of Cape Town using a validated LC-MS/MS assay. The lower limit of quantification (LLOQ) was 0.00781 mg/L. The inter-day accuracy ranged from 101% to 105%, and the precision (%CV) ranged from 3.3% to 4.6% during sample analysis.

Additional data were obtained from a 14 day Phase 2A early bactericidal activity (EBA) trial of clofazimine alone or in combination with bedaquiline, pretomanid and pyrazinamide.^12^ Clofazimine was administered as a loading dose of 300 mg daily on Days 1–3, followed by 100 mg daily on Days 4–14. PK sampling was performed pre-dose and 5 and 10 h after observed doses on Days 1, 2, 3 and 8; pre-dose, hourly up to 5 h, and at 10, 16 and 24 h after observed doses on Day 14, plus an additional sample at Day 28 (2 weeks after clofazimine discontinuation). Bioanalysis for drug plasma concentrations was conducted by PRA (Lenexa, KS, USA) using liquid–liquid extraction and ultra-performance LC-MS/MS. The LLOQ was 0.004 mg/L; inter-day accuracy ranged from 98.5% to 103%, and the precision (%CV) ranged from 2.3% to 3.7% during sample analysis.

### PK analysis

Non-linear mixed-effects modelling was used to analyse clofazimine concentrations from both clinical cohorts simultaneously using NONMEM version 7.4.3,^13^ Pirana, Perl-speaks-NONMEM (PsN) version 4.9.0 and Xpose4.^14^ We tested one-, two- and three-compartment disposition models. For the absorption process we tested first-order absorption with and without lag, saturable absorption, sum of inverse Gaussians^15^ and chain of transit compartments.^16^ Values below the LLOQ were excluded from the dataset.^13^

Allometric scaling was applied on all disposition parameters to account for the effect of body size descriptors, including total body weight (TBW), fat-free mass (FFM) and body fat.^17^ Individual values of FFM were derived from observed TBW, height and sex using a validated formula (provided in the Supplementary data, available at *JAC* Online).^18^ Fat mass was obtained as the difference between TBW and FFM. Age, sex, race, HIV status, clofazimine dose, treatment arm, duration on treatment and use of lopinavir/ritonavir (a strong CYP3A4 and P-glycoprotein inhibitor) were evaluated as additional covariates. Sampling importance resampling (SIR)^19^ was used to assess the robustness of the final parameter estimates and to obtain 95% CIs. Details of the modelling approach are provided in the Supplementary data.

### Simulations

Using the final model, we simulated time to steady-state for typical body size descriptors of female and male participants observed in our cohort. The final model was also used to simulate different dosing regimens to shorten the time to average daily exposure above a putative target concentration of 0.25 mg/L extrapolated from a murine model^10^ while avoiding excessive peak values above those attained at steady-state with standard dosing to reduce risk of QT prolongation. Various loading doses and durations were explored, including 300 mg daily for 1–2 weeks and 200 mg daily for 2–8 weeks. For the latter simulations, characteristics of a typical TB patient weighing 56 kg and different proportions of fat mass (13%, 20% and 34%) to reflect body size composition in the average TB patient and genders in the cohort, including random interindividual variability, were repeated 10 000 times. Simulations were performed using PsN 4.9.0, Berkeley Madonna version 9.1.19, and NONMEM version 7.4.3.

## Results

### Demographics and clinical profile

Seventy-nine participants on clofazimine-based regimens with complete 6 month follow-up data were included from the PROBeX observational cohort, in addition to 60 participants from the EBA trial. Overall, median age was 31.0 years (IQR 24.0–39.5), 68 (48%) were female and 54 (39%) were HIV infected. Median weight was 55.1 kg (IQR 48.1–60.9) and percentage body fat was significantly higher in women [median 33.8% (IQR 30.9–37.7) versus 13.9% (IQR 12.0–16.6) in men] (Table 1 and Figure S1).

**Table 1.** Baseline participant characteristics

Variable	PROBeX^a^	Phase 2A trial	Total
	(*n *=* *79)	(*n *=* *60)	(*n *=* *139)
Age, years	32.5 (26.5–40.0)	29.5 (23.0–39.0)	31.0 (24.0–39.5)
Females, *n* (%)	45 (57)	23 (38)	68 (49)
Ethnicity, *n* (%)			
black	53 (67)	29 (48)	84 (60)
mixed race	24 (30)	31 (52)	55 (39)
white	2 (3)		2 (1)
Weight, kg	54.0 (48.0–60.0)	55.9 (50.0–61.7)	55.3 (48.1–61.5)
males	54.2 (50.0–58.4)	57.9 (50.4–62.2)	56.0 (50.0–60.4)
females	53.2 (45.7–63.2)	54.3 (47.5–59.7)	53.9 (46.5–61.5)
FFM, kg	40.3 (35.0–46.2)	44.9 (37.1–50.1)	42.0 (36.3–48.3)
males	46.6 (43.4–49.9)	49.1 (45.1–52.9)	48.0 (44.1–51.2)
females	35.5 (32.0–39.8)	35.5 (32.9–38.9)	35.4 (32.1–39.7)
Body fat, kg	11.2 (7.91–19.3)	11.0 (7.2–17.5)	11.2 (7.6–18.3)
males	7.60 (5.75–9.48)	8.07 (5.59–11.0)	7.74 (5.69–9.77)
females	18.1 (14.1 –24.0)	18.7 (14.7–21.1)	18.5 (14.1–22.1)
Percentage body fat, %^b^	25.9 (14.4–34.0)	17.1 (12.7–32.8)	20.3 (13.9–33.7)
males	13.8 (12.2–16.1)	14.3 (11.0–17)	14.0 (11.9–16.4)
females	33.8 (30.6–38.1)	33.9 (32.1–36.0)	33.8 (30.9–37.7)
Height, cm	164 (157–168)	167 (161–173)	164 (158–172)
BMI, kg/m^2^	20.0 (18.0–22.5)	20.0 (17.9–22.0)	20.0 (18–21.9)
HIV positive, *n* (%)	49 (62)	5 (8)	54 (39)
CD4 count, cells/mm^3^	196 (96–437)	715 (515–893)	540 (268–831)
ART	40 (82)	0	40 (74)
lopinavir/ritonavir	11 (22)	0	11 (20)
*M. tuberculosis* resistance profile, *n* (%)			
drug susceptible	0	60 (100)	60 (43)
pre-XDR (Inj-R)	14 (18)		14 (10)
pre-XDR (FQ-R)	39 (49)		39 (28)
XDR	26 (33)		26 (19)
Serum creatinine, μmol/L	57.0 (48.5–68.0)	61 (51.4–70.8)	58.0 (50.5–69.3)
Duration on clofazimine, days	100 (55–182)	7.5 (4–11)	94 (48–180)

Data are median (range) unless specified otherwise.

Inj-R, injectable resistant; FQ-R, fluoroquinolone resistant.

a Median was imputed for missing values in continuous variables; 3 for age, 1 for weight and height, and 2 for serum creatinine.

b Calculated as fat mass/total body weight.

### Model development and PK parameters

Clofazimine concentrations from 1570 plasma samples (*n *=* *139 participants) were included in the analysis: 367 observations from three sampling visits in the PROBeX cohort (including intensive sampling from 22 participants) and 1203 observations from sampling over the 14 day EBA trial. Six samples had concentrations below the LLOQ and were excluded from the analysis. Twenty-five sparse samples from PROBeX participants with poor treatment adherence or missing dosing history had concentrations significantly lower than predicted on visual inspection and were also excluded.

**Figure 1.**
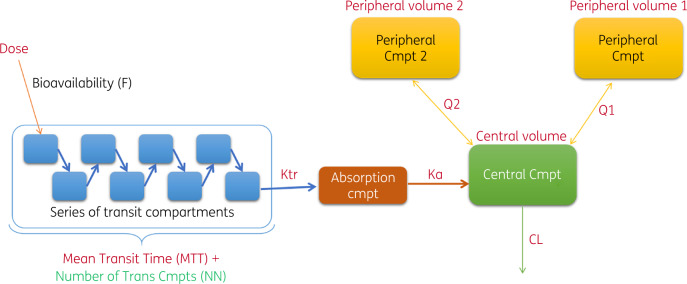
Schematic of the final clofazimine population PK model. The dose is assumed to go through a series of transit compartments, where NN describes the number of transit compartments and the transit rate constant (Ktr) the transfer rate, calculated as NN + 1 divided by the mean transit time (MTT). Drug is then absorbed into the central compartment (representing the plasma concentration of clofazimine), described by the absorption rate constant (Ka). Bidirectional equilibration (Q1 and Q2) occurs with two peripheral tissue compartments, deep (V_p1_) and shallow (V_p2_), while drug is eliminated from the central compartment with first-order kinetics (CL). Cmpt, compartment. This figure appears in colour in the online version of *JAC* and in black and white in the print version of *JAC*.

**Figure 2.**
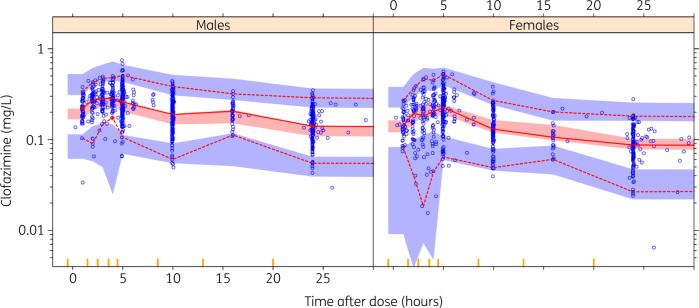
Prediction-corrected visual predictive check for pooled clofazimine concentration versus time (time after dose), stratified by sex. Circles represent original data, dashed and solid lines are the 5th, 50th and 95th percentiles of the original data, and the shaded areas are the corresponding 95% CI for the same percentiles, as predicted by the model. Vertical yellow lines on the *x*-axis represent bins for sampling timepoints. An appropriate model is expected to have most observed percentiles within the simulated CIs. This figure appears in colour in the online version of *JAC* and in black and white in the print version of *JAC*.

Clofazimine PK were best characterized by a three-compartment disposition model with first-order elimination and absorption in transit compartments (Figure 1). Size descriptors were normalized to median population values. The best size descriptors for scaling of disposition parameters were: TBW for total (CL) and intercompartmental (Q1, Q2) clearance and volume of distribution in the ‘shallow’ peripheral compartment (V_p2_); FFM for central volume (V_c_); and fat mass for volume of distribution in the ‘deep’ compartment (V_p1_). The latter parameter resulted in the largest change in objective function value, indicating a significant effect, when included in the model. Age, sex, race, HIV status, duration of treatment and treatment arm were not identified as influential covariates according to stratified visual predictive checks and change in objective function, performed using the final model. The model detected an effect of lopinavir/ritonavir on clofazimine exposure, leading to higher bioavailability; however, the estimate was not sufficiently robust in terms of change in objective function to be included in the final model. Residual unexplained variability was best described by a combined model utilizing both additive and proportional components, with separate additive error estimates for the sparse dataset from PROBeX (due to uncertainty in self-reported dosing times and adherence). A prediction-corrected visual predictive check suggested adequate fit for the pooled data (Figures 2 and S2).

The final model parameter estimates are summarized in Table 2. The typical value for clearance (CL/F) was 11.5 L/h, and 10 500 L for the ‘deep’ peripheral volume. Women had much larger ‘deep’ peripheral volume (13 700 L for a typical woman versus 5720 L for a typical man) explained by significantly higher body fat percentage compared with men. The model detected an increase in bioavailability over the first few days of treatment, from a baseline of 68% to 95% of the reference value by the fourth dose. Because participants received a larger daily dose (300 mg versus 100 mg) in the first 3 days of the EBA trial, dose effect was investigated as a potential explanation, but a time-dependent exponential increase model (described in the Supplementary data) fitted the data better, even after testing several alternative absorption processes.

**Table 2.** Final population PK model parameters

Parameter description	Typical value (95% CI)^a^
Clearance, (L/h)^b^	11.5 (10.5–12.5)
Central volume of distribution, V_c_ (L)^b^	262 (178–375)
Intercompartmental clearance, Q1 (L/h)^b^	56.3 (49.6–62.6)
Peripheral volume 1, V_p1_ (L)^b^	10 500 (9320–11 600)
Intercompartmental clearance, Q2 (L/h)^b^	86 (74.6–99.5)
Peripheral volume 2, V_p2_ (L)^b^	889 (696–1070)
Absorption mean transit time (h)	1.41 (1.10–1.67)
Number of transit compartments (NN)	4.75 (3.01–7.65)
Absorption rate constant, Ka (1/h)	0.209 (0.175–0.261)
Relative bioavailability baseline	0.685 (0.615–0.771)
Bioavailability, F	1 [FIXED]
Proportional error (%)	11.4 (10.8–12.1)
Additive error (mg/L)^c^	0.00156 [FIXED]
Additive error (PROBeX sparse data) (mg/L)	0.0905 (0.077–0.107)
P-glycoprotein inhibition half-life (days)^d^	1.44 (0.683–2.85)
Between-subject variability (%)^e^	
clearance	25.6 (17–33.5)
central volume	23.5 (8.39–35.1)
peripheral volume 1	29.6 (20.4–38.1)
peripheral volume 2	54.6 (42.3–73.9)
bioavailability	30.1 (25.2–35.5)
Between-occasion variability (%)^e^	
absorption mean transit time	46.6 (38.1–56.9)
absorption rate constant	32.6 (27.1–38)
bioavailability	35.4 (31.8–39.6)
Terminal half-life (days)^f^	
median patient	34.2
female	49.5
male	21.8

a 95% CI obtained with the sampling importance resampling technique using PsN software.

b Allometric scaling used for CL, V_c_, V_p1_, V_p2_, Q1 and Q2; typical values reported for the median weight (55 kg), FFM (42 kg) and fat mass (13 kg) as reported in Table 1.

c Estimate of the additive error was not statistically significant from the lower bound (LLOQ/2) and was thus fixed to that value.

d Inhibitory effect of clofazimine on P-glycoprotein using an exponential maturation function (described in the Supplementary data).

e Between-subject variability and between-occasion variability were assumed to be log-normally distributed and reported as approximate %CV.

f Derived parameters outside the estimation software: calculated for the typical male and female median values as reported in Table 1.

**Figure 3.**
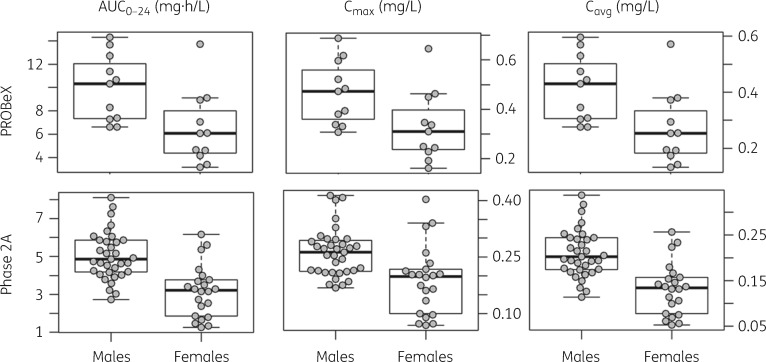
Box and whisker plots showing secondary model-derived non-compartmental parameters, stratified by sex. The dots represent individual values; the central lines in boxes represent median values; upper and lower horizontal lines of boxes are 75th and 25th percentiles, respectively; and whiskers are 2.5th and 97.5th percentiles. *n *=* *22 for PROBeX, sampled at ∼2 months; *n *=* *57 for the Phase 2A trial, sampled at Day 14.

Model-predicted clofazimine exposure [*C*_max_, AUC_0–24_ and average daily concentration (*C*_avg_)] was higher at 2 months compared with 14 days, reflecting clofazimine accumulation over time. Estimated plasma exposures were lower in women (Table 3 and Figure 3).

**Table 3.** Model-predicted secondary PK parameters from rich sampling occasions

Study	Females	Males	Total
PROBeX (Month 2)			
*C*_max_ (mg/L)	0.310 (0.161–0.646)	0.473 (0.307–0.688)	0.363 (0.161–0.688)
AUC_0–24_ (mg·h/L)	6.08 (3.17–13.7)	10.3 (6.61–14.3)	7.33 (3.17–14.3)
*C*_avg_ (mg/L)	0.254 (0.132–0.572)	0.430 (0.275–0.596)	0.305 (0.132–0.596)
Phase 2A (Day 14)			
*C*_max_ (mg/L)	0.199 (0.0687–0.403)	0.263 (0.168–0.413)	0.218 (0.0687–0.4130)
AUC_0–24_ (mg·h/L)	3.22 (1.26–6.16)	4.87 (2.73–8.60)	4.2 (1.26-8.60)
*C*_avg_ (mg/L)	0.134 (0.0523–0.257)	0.203 (0.114–0.358)	0.177 (0.0578–0.3580)

Data are given as median (IQR).

*n *=* *22 for PROBeX, sampled at ∼2 months; *n *=* *57 for the Phase 2A trial, sampled at Day 14.

### Simulations

**Figure 4.**
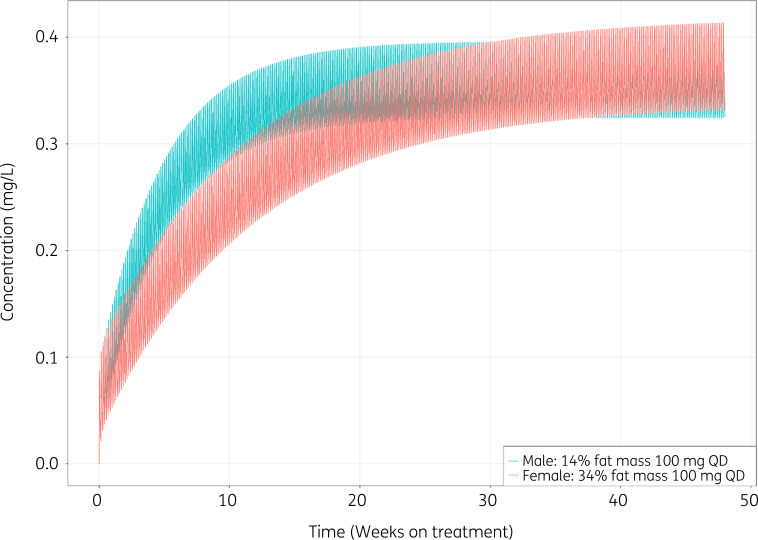
Predicted clofazimine concentrations at steady-state with standard dosing (100 mg daily), stratified for typical male/female participants in the cohort. This figure appears in colour in the online version of *JAC* and in black and white in the print version of *JAC*.

Median terminal elimination half-life was estimated at 34.2 days and was significantly longer for women (49.5 days versus 21.8 days for men), resulting from differences in body composition. Consequently, the median time to steady-state (∼5 times the terminal half-life), which was 150 days overall, was much shorter for men (105 days versus 230 days for women) (Figure 4).

**Figure 5.**
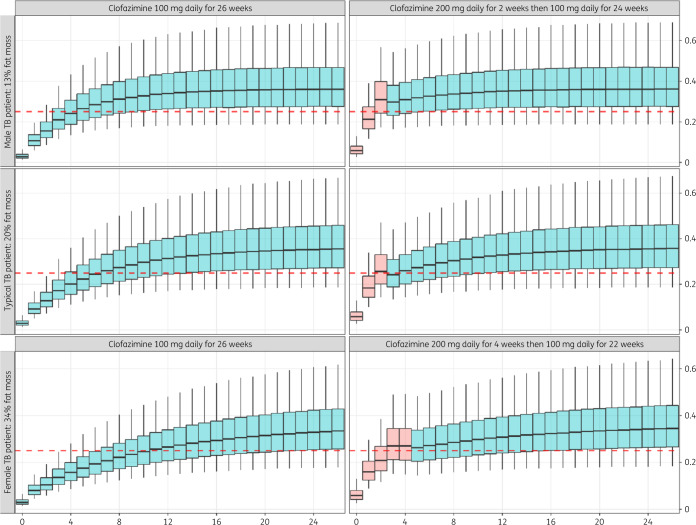
Simulated exposures with standard dosing and loading dose for typical male, typical female and all TB patients in the cohort. Dashed lines represent the suggested target concentration (0.25 mg/L); the central lines in boxes represents median values; upper and lower horizontal lines are 75th and 25th percentiles, respectively; and whiskers are 2.5th and 97.5th percentiles. Orange shaded boxplots represent the loading dose period. Note, time is truncated at 26 weeks for improved visualization. This figure appears in colour in the online version of *JAC* and in black and white in the print version of *JAC*.

Simulations of a 300 mg loading dose for either 1 or 2 week durations predicted peak concentrations (*C*_max_) that exceeded those estimated at steady-state, a potential safety concern, particularly for QT prolongation (Figure S3). A simulated schedule of 200 mg daily loading dose given for the initial 2 weeks of therapy followed by standard dosing (100 mg daily) predicted average daily plasma concentrations above the *Mycobacterium tuberculosis* WT MIC value (0.25 mg/L) 37 days earlier compared with standard dosing for a typical TB patient in our cohort. For typical male patients, with 13% body fat, the 2 week loading dose achieved a 21 day reduction in time to target concentration. However, female patients, who had an average of 34% body fat, required a longer loading period: a simulation of 4 weeks’ loading led to target attainment 56 days earlier compared with standard dosing in women (Figure 5). Predicted median *C*_max_ with use of this loading dose was 0.277 mg/L at the end of the loading period, compared with median *C*_max_ 0.343 mg/L at steady-state.

## Discussion

Clofazimine PK have not been adequately studied and data from TB patients are especially scarce. Using data from a prospective observational study and an EBA trial, we developed a population PK model that describes the compartmental kinetics and accumulation of clofazimine in South African TB participants with 39% prevalence of HIV co-infection. Clofazimine disposition was strongly influenced by body fat percentage, thus resulting in initially lower plasma exposure amongst women. We simulated a loading dose that would achieve steady-state faster without increasing expected peak concentrations, hence limiting the likelihood of added toxicity. These findings have implications for clofazimine dosing in TB.

Our model-predicted PK parameters confirm a large volume of distribution (∼10 000 L) and long terminal half-life of ∼30 days, which is consistent with known pharmacological properties of clofazimine.^7^ Clofazimine is highly lipophilic^20^ and distributes widely into fatty tissues;^21^^,^^22^ murine experiments have demonstrated that clofazimine primarily accumulates in reticuloendothelial tissues with slow decline in serum and organs after discontinuation (ranging from 45 to 70 days).^6^^,^^10^^,^^23^^,^^24^ These murine observations were recapitulated in single- and multiple-dose healthy volunteer studies which applied exponential functions to model biphasic elimination of a 50 mg daily dose; estimated time to steady-state was ∼60 days with marked accumulation over time.^25^ The widely cited terminal elimination half-life of 70 days in humans was derived from urine clofazimine concentrations in leprosy patients and healthy volunteers over four decades ago.^26^ The complex PK of clofazimine,^25^ with multiphase disposition and very long terminal half-life, are difficult to accurately characterize using non-compartmental analysis.^11^ There have been few attempts to model clofazimine PK to accurately estimate disposition in people. An unpublished two-compartment disposition model using data from leprosy patients and healthy volunteers with variable dosing schedules estimated a peripheral volume of around 4000 L and a half-life of 15 days.^27^ Another study, applying a one-compartment model to quantify the effect of food on clofazimine bioavailability in healthy volunteers, reported a mean volume of distribution of 1470 L, although the authors were unable to estimate the terminal elimination phase due to limited assay sensitivity.^28^ The much higher volumes predicted by our model could reflect important differences in the physiology of TB patients, as well as the influence of population-specific covariates.

The pooled data and observed variance in our cohort were well described by a three-compartment structural model that accounted for the complex multiphase disposition.^25^ Lopinavir/ritonavir use was associated with a moderate increase in clofazimine exposure. Clofazimine metabolism is poorly characterized; one study found clofazimine to be a P-glycoprotein substrate on *in vitro* screening^9^ and thus a potential victim of P-glycoprotein inhibition by HIV protease inhibitors. This has been an inconsistent finding,^8^ and formal drug–drug interaction studies are needed to investigate the effect of co-administration with lopinavir/ritonavir on clofazimine exposure and toxicity.

Body composition explained our key finding of major sex differences in clofazimine plasma exposure. In our combined cohorts, women had a higher proportion of body fat and consequently a larger peripheral volume of clofazimine distribution compared with men. Because PK sampling occurred before steady-state attainment while the drug was still accumulating in peripheral tissues, women from both cohorts had much lower observed clofazimine plasma concentrations and derived exposure parameters (AUC_0–24_, *C*_max_ and *C*_avg_), which is in agreement with reported findings from non-compartmental analyses of the EBA trial^12^ and the observational PROBeX study.^29^ The clinical consequence of larger peripheral volumes is extended terminal half-life, prolonging the predicted number of repeated daily doses necessary to achieve clofazimine steady-state in women, demonstrated by our simulations in Figure 4.

Average clofazimine plasma concentrations measured before steady-state were much lower than the recommended critical concentration of 1 mg/L and published MIC distributions in drug-resistant *M. tuberculosis* strains.^30^ Clofazimine is highly protein bound,^6^^,^^31^ resulting in free plasma drug concentrations below the MIC, indicating a potentially suboptimal antibacterial effect. However, clofazimine has been shown to partition in the cellular rim of explanted lung granulomas, achieving much higher intracellular concentrations relative to plasma.^32^ Tissue accumulation and the long terminal elimination half-life of clofazimine may contribute to efficacy and treatment shortening as a consequence of high site-of-disease concentrations despite failure to exceed the MIC in plasma.^32^^,^^33^ Clofazimine has no discernible EBA at 14 days^12^ and consistently demonstrates delayed and concentration-dependent activity in murine models.^6^^,^^10^ Simulations based on sparse data from two Korean MDR-TB patients on standard doses predicted attainment of clofazimine concentration above the efficacy target in cellular lesion compartments at steady-state.^33^ While steady-state tissue exposures are likely to exceed bactericidal concentrations (0.25 mg/L)^34^ and contribute to sterilizing activity against intracellular bacilli, early treatment efficacy could be optimized using a loading dose to achieve steady-state more rapidly.

There is no established clinical PK/PD index for clofazimine efficacy in TB, precluding empirically based dosing simulations. In a murine model, clofazimine was shown to contribute sustained antimycobacterial activity for up to 6 weeks after discontinuation, and this ‘post-antibiotic effect’ was associated with plasma concentrations above 0.25 mg/L;^10^ we therefore selected average daily concentration above this value as the target efficacy parameter for our dosing simulations. The relationship between clofazimine exposure and toxicity (QT prolongation and skin changes) is unknown. Peak serum drug concentrations generally correspond to peak effect on QT interval, and our loading dose simulations aimed to balance more rapid attainment of efficacy target concentration with anticipated effects of higher initial *C*_max_ on QT prolongation. The principles guiding selection of dosing strategies were thus to: (i) avoid substantially higher predicted *C*_max_ after the loading period than average steady-state peak concentrations to reduce the risk of serious QT prolongation; (ii) achieve shorter time to average daily concentrations exceeding the efficacy target; and (iii) use a simple dosing regimen with once-daily administration. A dosing schedule of 200 mg for 2 or 4 weeks depending on body fat percentage followed by 100 mg daily achieved these objectives and requires further investigation in future clinical trials to delineate the impact on important endpoints and treatment shortening.

As with all modelling exercises, our analysis has limitations. While the model presented here was able to describe complex data with relatively few parameters, mechanistic assumptions were based on very limited knowledge of clofazimine metabolism and may have limited predictive ability outside the range of observed data.^35^ There was also some uncertainty in the data, particularly around accuracy of dosing times and adherence relating to sparse PK sampling in the PROBeX cohort; we accounted for this by introducing a separate additive error estimate to the model. Furthermore, we based our model on pooled data from two separate clinical cohorts and drug assays from different laboratories, which may add unexplained variability. Our model detected a moderate increase in clofazimine bioavailability over the first few days of therapy, which we attribute to putative autoinhibition of intestinal P-glycoprotein. However, this finding could be due to the limits of model extrapolation: EBA trial participants received a larger dose for 3 days and there was a high degree of variability in bioavailability. Although time-dependent rather than dose-dependent absorption describes the data better, dose-dependent saturation of absorption remains a possibility. Finally, assumptions for our dosing simulations were made in the absence of clofazimine PK/PD data from TB patients and reflect theoretical rather than empirically based targets.

In conclusion, we successfully developed a clofazimine population PK model for South African TB participants, describing massive peripheral distribution volume and prolonged terminal elimination. Clofazimine disposition was strongly influenced by body fat content, resulting in lower plasma exposure among women. The clinical consequences are unknown but clofazimine may require dose individualization at extremes of body composition to optimize use. A 2 week loading dose may support treatment shortening by enabling more rapid attainment of efficacy targets within a safe window of peak concentrations in this population; longer loading periods may be required in patients with high fat mass. This needs to be evaluated in clinical trials. Dose optimization of clofazimine is a research priority in TB, and our model is a necessary first step to understanding concentration–response relationships of this key anti-TB drug.

## Funding

S.W. is supported by the European & Developing Countries Clinical Trials Partnership (grant no. CDF1018), Wellcome Trust (grant no. 203135/Z/16/Z) and National Institutes of Health [NIH: K43TW011421, (PI Wasserman)]. J.C.M.B. is supported by the National Institutes of Health [R01AI114304 (PI Brust), P30AI124414 (PI Goldstein) and UL1TR001073 (PI Shamoon)]. The National Research Foundation provided funding to P.D. (grant no. 109056). M.T.A., P.D. and E.S. were supported by the Swedish Foundation for International Cooperation in Research and Higher Education (STINT) jointly with the South African National Research Council, National Research Foundation (NRF) (NRF grant no. 101575). N.R.G. is supported by the NIH [K24AI114444 (PI Gandhi), U19AI111211 (MPI Ernst/Blumberg)]. Gr.M. was supported by the Wellcome Trust (098316 and 203135/Z/16/Z), the South African Research Chairs Initiative of the Department of Science and Technology and National Research Foundation (NRF) of South Africa (grant no. 64787), NRF incentive funding (UID: 85858) and the South African Medical Research Council through its TB and HIV Collaborating Centres Programme with funds received from the National Department of Health (RFA# SAMRC-RFA-CC: TB/HIV/AIDS-01–2014). The 2 week bactericidal activity study was sponsored by TB Alliance (Global Alliance for TB Drug Development) with support from the Bill and Melinda Gates Foundation, the US Agency for International Development, UK Department for International Development, Irish Aid and Australia Department of Foreign Affairs and Trade.

## Transparency declarations

None to declare.

## Supplementary data

Figures S1 to S3 are available as Supplementary data at *JAC* Online.

## Supplementary Material

dkaa310_supplementary_data
