# Supplementary material for: Clofazimine pharmacokinetics in patients with TB: dosing implications
Source: J Antimicrob Chemother. 2020 Aug 3;75(11):3269–77. doi: 10.1093/jac/dkaa310 (PMC7566350; doi:10.1093/jac/dkaa310)
Supplement: dkaa310_supplementary_data [file dkaa310_supplementary_data.docx]

**Supplementary data**


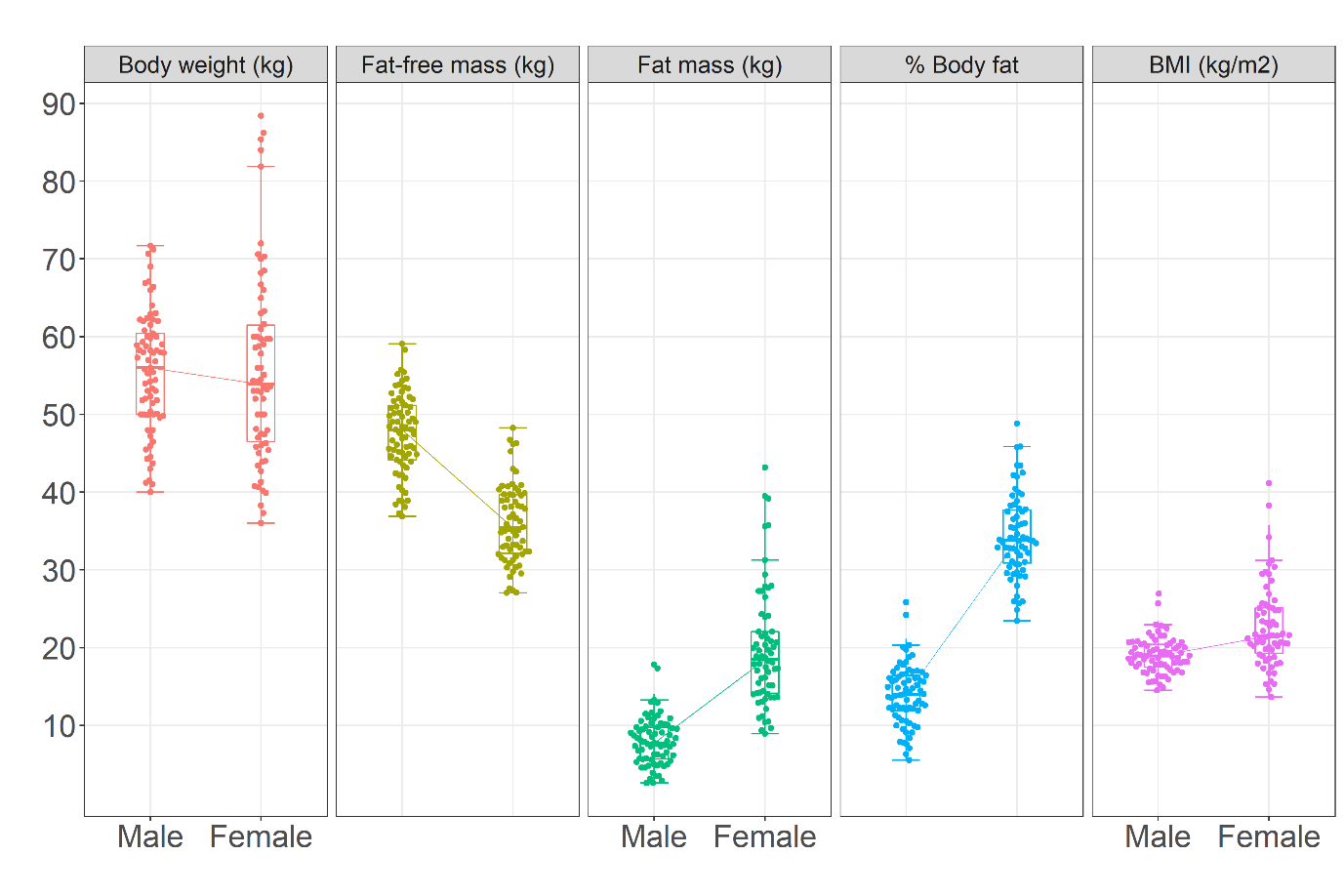


**Figure S1**

**Distributions of body composition components, stratified by sex**


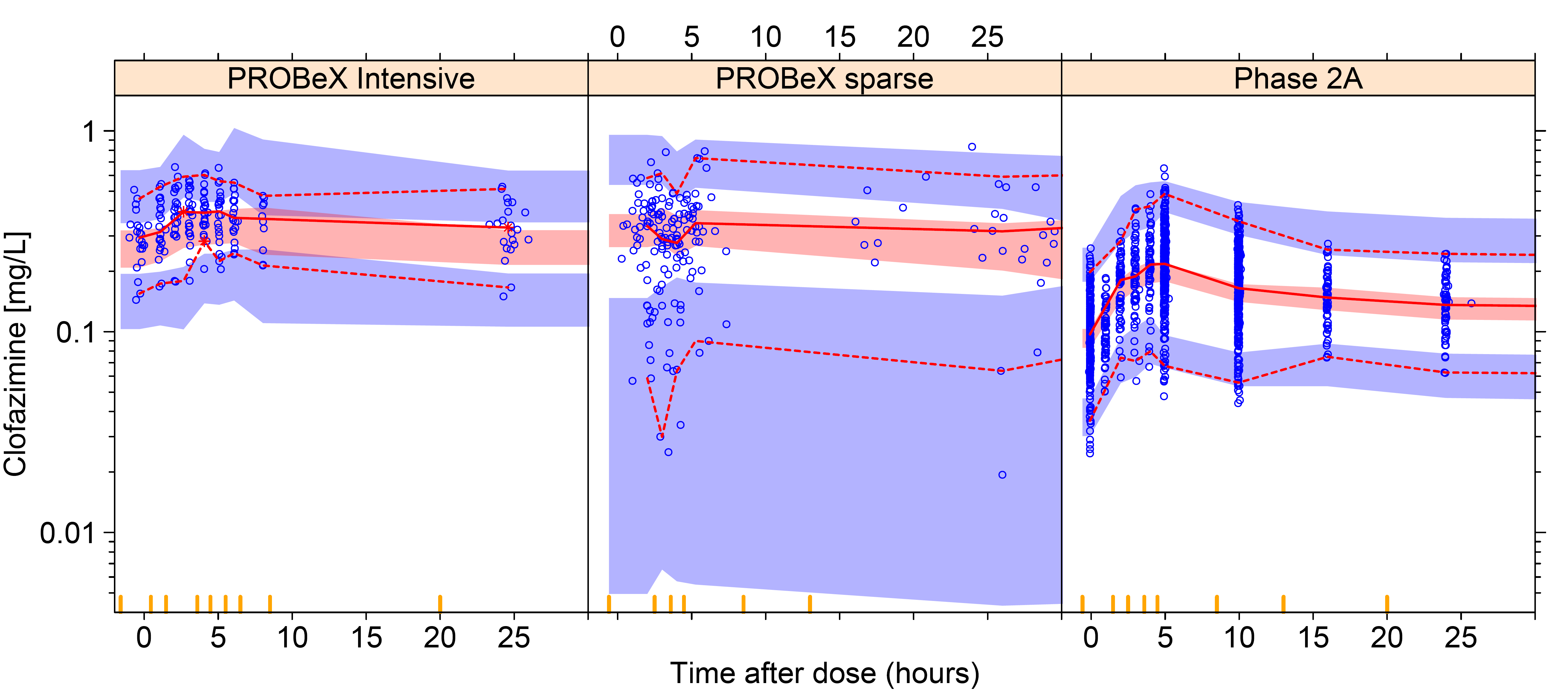


**Figure S2**

**Prediction-corrected visual predictive check (pc-VPC) for pooled clofazimine concentration versus time (time after dose), stratified by dataset.**

Circles represent original data, dashed and solid lines are the 5th, 50th, and 95th percentiles of the original data, while the shaded areas are the corresponding 95% confidence intervals for the same percentiles, as predicted by the model. Vertical yellow lines on the x-axis represent bins for sampling timepoints. An appropriate model is expected to have most observed percentiles within the simulated confidence intervals.

**
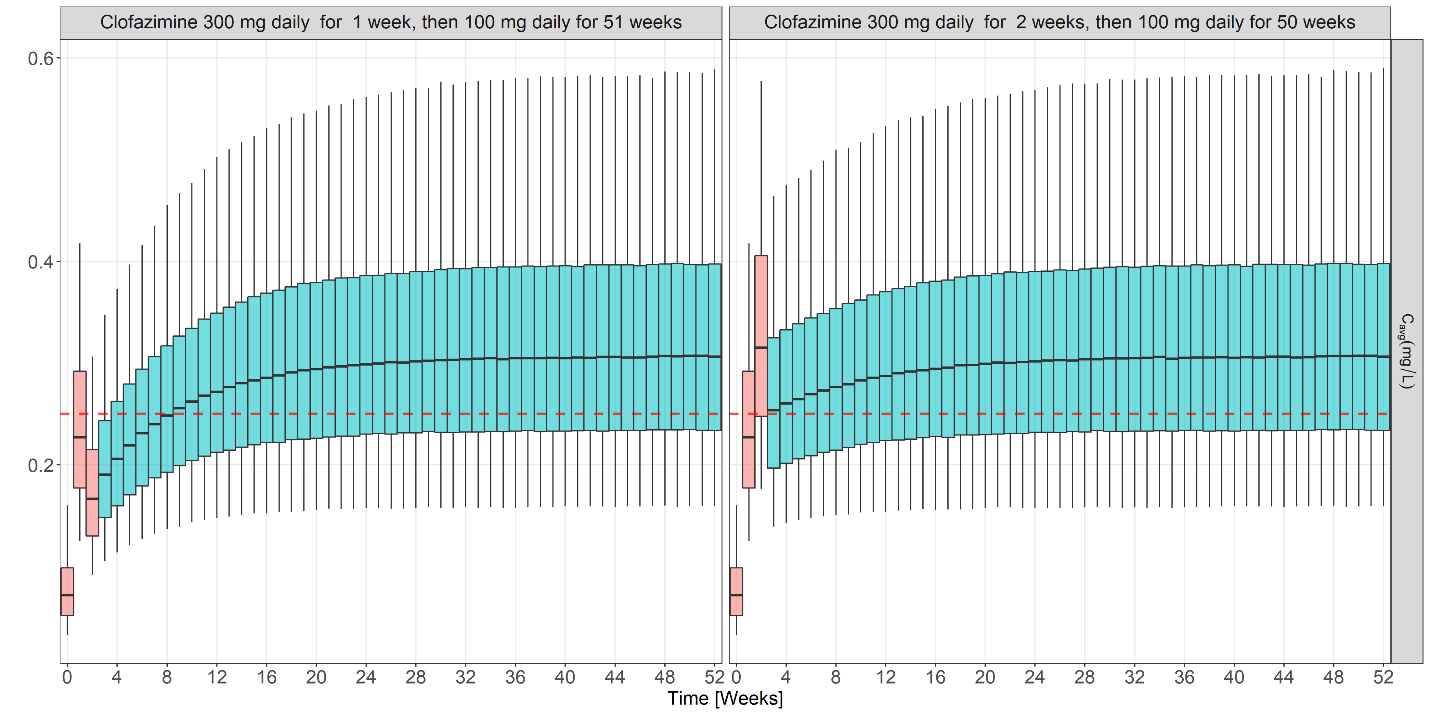
**

**Figure S3. Simulated peak exposures with different loading doses for a typical patient Weight :70 kg; fat-free mass: 56 kg; fat mass:14 kg**

Dashed line represents the suggested concentration target (0.25 mg/L). whiskers are 2.5^th^ and 97.5^th^ percentiles. Orange shaded boxplots represent first two weeks of treatment.

**Population PK modeling**

Between-subject and -occasion random effects were included on the PK parameters assuming log-normal distribution; a combined additive and proportional structure described the residual unexplained variability, with the additive component of the error constrained to be least 20% of lowest level of quantification (LLOQ). Values below LLOQ (BLQ) were excluded from the dataset based on the M1 method (13). We also dropped observations that were lower than predicted based on visual inspection and confirmed poor treatment adherence.

We tested total body weight (TBW), fat-free mass (FFM), and body fat proportion as body size descriptors. Individual values of FFM were derived from observed TBW, height, and sex using a validated formula (16).Fat mass (FAT) was estimated from the difference between TBW and FFM. Allometric scaling was applied on all disposition parameters to adjust for the effect of body size; scaling exponents were fixed to 0.75 for clearance parameters, and 1 for volumes (17):

$Allomt_{WT}= \left( \frac{TBW}{TBW_{median}} \right)^{\theta}$ $Allomt_{FFM}= \left( \frac{FFM}{FFM_{median}} \right)^{\theta}$ $Allomt_{FAT}= \left( \frac{FAT}{FAT\_median} \right)^{\theta}$

θ was fixed to 0.75 for clearance parameters (CL, Q1, Q2) and to 1 for volume parameters (Vc, Vp1, Vp2)

$$FFM=\frac{WHS_{max} .{Ht}^{2}.TBW}{Ht^{2}.WHS_{50}+TBW}$$

WHS_max_ is 42.92 and WHS_50_ is 30.93 in males and 37.99 and 35.98 in females, Ht is height in meters and TBW is total body weight, the extra fat weight (FAT) is obtained from the difference between total body weight and fat free mass.

The development of the model and the inclusion of covariates were based on physiological plausibility, inspection of diagnostic plots, including visual predictive checks (VPCs) (18), and decreases in the NONMEM objective function value (OFV), which was assumed to follow a Chi-squared (χ2) distribution. The statistical significance cut-off for an additional degree of freedom (inclusion of one additional parameter) was an OFV drop of at least 3.84 points, corresponding to p < 0.05. Covariates were tested in a stepwise fashion in order of importance determined by the largest significant decrease in OFV.

We modelled the inhibitory effect of clofazimine on P-glycoprotein using an exponential maturation function which was added on bioavailability as follows:

$$TVBIO = (BIO\_BS+(BIO\_SS-BIO\_BS)\cdot(1- e^{(-ln\left( 2 \right)\cdot\frac{DUR}{Inib_{50}})}))$$

The function estimated the effect of treatment duration (DUR) from a relative baseline bioavailability (BIO_BS) to an “effect steady-state” (BIO_SS) with inhibition half-life, Inhib_50_.

**NONMEM control stream of the final model**

;Model desc: Clofazimine Pop Pk model in South African TB patients

;Settings for the memory of NONMEM

$SIZES PD=-1000 LVR=-150 LTH=-200 MAXFCN=10000000 LNP4=-150000

$PROBLEM --------------------------------

$INPUT --------------------------------

$DATA -------------------------------- IGNORE=#

$ABBREVIATED COMRES=2

$SUBROUTINE ADVAN13 TRANS1 TOL=9 ATOL=9 SSTOL=6 SSATOL=6

$MODEL NCOMPARTMENTS=5 COMP=(ABSORB DEFDOSE)

COMP=(CENTRAL DEFOBSERVATION) COMP=(PERI) COMP=(PERI2)

COMP=("AUC",INITIALOFF)

;initializing population parameters

;DAT type = 1 for PRobex Intesnive data, 2 for Probex Sparse data and 3 for Phase 2A dataset

$THETA ------------------------------

--------------------------------

;--Omega---------------------------------

$OMEGA --------------------------------

--------------------------------

;------------------------------------------------------------

$SIGMA 1 FIX ; SIGMA

;********************************************************************DONE

$PK

IF (NEWIND.NE.2) B_DUR = DUR_CFZ

DELTA_DUR = DUR_CFZ - B_DUR

;ADDING DOSE VARIABLE

IF (CFZ_DZ.EQ.100) THEN

DOZ = 0

ELSE

DOZ = 1

ENDIF

;Adding allometric scalling

HTM = HT/100 ;rescaling HT to m units

;Female has value of sex 2 and male value of sex 1

IF (SEX.EQ.2) THEN

WHSMAX=37.99

WHS50=35.98

ELSE ;males

WHSMAX=42.92

WHS50=30.93

ENDIF

;---------------------------------------------------------

HTM2 = HTM**2

FFM = (WHSMAX*HTM2*WT)/(WHS50*HTM2+WT)

FAT = WT-FFM

FAT_PER = (FAT/WT)*100

;-------Typical values of WT,FFM and FAT

TVWT = --------------------------------

TVFAT = --------------------------------

TVFFM = --------------------------------

;---------Allometric scaling and covariates

ALLMCL_WT = (WT/TVWT)**0.75

ALLMV_WT = (WT/TVWT)

ALLMCL_FAT = (FAT/TVFAT)**0.75

ALLMV_FAT = (FAT/TVFAT)

ALLMCL_FFM = (FFM/TVFFM)**0.75

ALLMV_FFM = (FFM/TVFFM)

;---------------------------------

BIOBS=THETA(4) ; BIO baseline=0

BIOSS=THETA(16) ; BIO STEADY STATE

IND50=THETA(17) ; HALF LIFE

;Population parameters

TVV3 = THETA(1)*ALLMV_FAT

TVQ = THETA(2)*ALLMCL_WT

;------------------------------------

TVKA = THETA(3)

IF (DAT_TYPE.EQ.3) THEN

TVBIO = (BIOBS+(BIOSS-BIOBS)*(1- EXP(-LOG(2)*DELTA_DUR/IND50)))

ELSE

TVBIO = BIOSS

ENDIF

;THETA(5) ; PROPR ERROR

;THETA(6) ; ADD ERROR

TVMTT = THETA(7)

TVCL = THETA(8)*ALLMCL_WT

TVV = THETA(9)*ALLMV_FFM

TVNN = THETA(11)

TVQ2 = THETA(14)*ALLMCL_WT

TVV4 = THETA(15)*ALLMV_WT

;------------------------------------------------------------------------------

;Defining Between subjects' ETA's

BSVCL = ETA(1)

BSVV = ETA(2)

BSVVP = ETA(3)

BSVQ = ETA(4)

BSVVP_2 = ETA(5)

BSVQ2 = ETA(6)

BSVKA = ETA(7)

BSVBIO = ETA(8)

BSVMTT = ETA(9)

;BETWEEN VISIT VAR ON CL

BOVCL = 0

IF (OCC==30) BOVCL = ETA(10)

IF (OCC==40) BOVCL = ETA(11)

IF (OCC==50) BOVCL = ETA(12)

IF (OCC==600.OR.OCC==60) BOVCL = ETA(13)

IF (OCC==700.OR.OCC==70) BOVCL = ETA(14)

;-----------------------------------------------------------------------------

BOVKA = 0

BOVMTT = 0

BOVBIO = 0

;Defining Between OCC variability;

;OCCASION 1-------------------------------------------------------------------

IF (OCC==1.OR.OCC==30) THEN ; to avoid having OCC without corresponding ETA's, either ; occasion from Probex or Phase 2A study

BOVKA = ETA(15)

BOVMTT = ETA(24)

BOVBIO = ETA(33)

ENDIF

;OCCASION 2-------------------------------------------------------------------

IF (OCC==2.OR.OCC==40) THEN

BOVKA = ETA(16)

BOVMTT = ETA(25)

BOVBIO = ETA(34)

ENDIF

;OCCASION 3--------------------------------------------------------------------

IF (OCC==3.OR.OCC==50) THEN

BOVKA = ETA(17)

BOVMTT = ETA(26)

BOVBIO = ETA(35)

ENDIF

;-----------------------------------

;OCCASION 10 INTENSIVE- PRE DOSE----------------------------------------------------------------

IF (OCC==10) THEN

BOVKA = ETA(18)

BOVMTT = ETA(27)

BOVBIO = ETA(36)

ENDIF

;OCCASION 20 INTENSIVE- INTENSIVE SAMPLES-------------------------------------------------

IF (OCC==20) THEN

BOVKA = ETA(19)

BOVMTT = ETA(28)

BOVBIO = ETA(37)

ENDIF

IF (OCC==600) THEN

BOVKA = ETA(20)

BOVMTT = ETA(29)

BOVBIO = ETA(38)

ENDIF

IF (OCC==60) THEN

BOVKA = ETA(21)

BOVMTT = ETA(30)

BOVBIO = ETA(39)

ENDIF

IF (OCC==700) THEN

BOVKA = ETA(22)

BOVMTT = ETA(31)

BOVBIO = ETA(40)

ENDIF

IF (OCC==70) THEN

BOVKA = ETA(23)

BOVMTT = ETA(32)

BOVBIO = ETA(41)

ENDIF

;-----------------------------------

;PARAMETERS

CL = TVCL*EXP(BSVCL+BOVCL) ; CLEARANCE

V = TVV*EXP(BSVV) ; CENTRAL VOL.

BIO = TVBIO*EXP(BSVBIO+BOVBIO) ; BIOAVAILABILITY

MTT =TVMTT*EXP(BSVMTT + BOVMTT) ; MTT TIME

V3 = TVV3*EXP(BSVVP) ; PERIPH VOL

Q = TVQ*EXP(BSVQ) ; INTER COMPT Q

V4 = TVV4*EXP(BSVVP_2) ; PERIPH VOL 2

Q2 = TVQ2 ; INTER COMPT Q2

NN = TVNN ; Number of Transit compartment

;--------------------------------------------------------------------------------------------------------------------

;re-parameterization

K = CL/V ;(rate constant of elimination)

K23 = Q/V ;(rate constant from central to peripheral 1)

K32 = Q/V3 ;(rate constant from peripheral 1 to central)

K24 = Q2/V ;(rate constant from central to peripheral 2)

K42 = Q2/V4 ;(rate constant from peripheral 2 to central)

KA = TVKA*EXP(BSVKA+BOVKA) ;ABS. RATE CONSTANT

F1 = 0 ; I need to set bioavailability in compartment 1 to 0 for transit absorption

KTR = (NN+1)/MTT

IF (NEWIND/=2.OR.EVID>=3) THEN ; new individual, or reset event

; The values read here will be stored in TDOS and PD in this very PK call.

TNXD=TIME ; Time of the dose

PNXD=AMT ; Amount. If it's zero, the DE is deactivated.

COM(1)=0 ; COMRESS 1

COM(2)=0 ; COMRESS 2

TIMEDOSE = TIME

AMOUNTDOSE = AMT

ENDIF

TDOS=TNXD ; This will either save here the temporary values if it's a new individual...

PD=PNXD ; ...or the values which were read one record ahead during the execution of the previous record.

IF(AMT.GT.0) THEN ; This reads one record ahead and stores the data to be used when running the following record

; IF(AMT.GT.0.AND.ALAG1.EQ.0) THEN ; Use this instead if there is ALAG, as it will also checks if the ALAG is not 0

TNXD=TIME

PNXD=AMT

ENDIF

IF (DOSTIM.GT.0) THEN ; This will account for the ADDL or lagged doses. It will overwrite the time, if it a non-event record

;.. DOSTIM>0: this call to PK occurs at a non-event dose time DOSTIM,i.e., at the time of an additional or lagged dose

TNXD=DOSTIM

PNXD=AMT

ENDIF

PIZZA = LOG(BIO*PD*KTR + 0.00001) - GAMLN(NN+1)

; INITIALIZING COMPARTMENTS TO SPEED UP COMPUATION

A_0(1) = 0.0001

A_0(2) = 0.0001

A_0(3) = 0.0001

A_0(4) = 0.0001

$DES

TEMPO = T-TDOS ; this is time after dose for the transit, it should always be >= 0

KTT = 0

DADT(1) = -KA*A(1)

IF(PD.GT.0.AND.TEMPO.GT.0) THEN ; This happens only if PD>0, so only if a dose has been ; detected

KTT = KTR*(TEMPO)

DADT(1) = EXP(PIZZA+NN*LOG(KTT)-KTT) -KA*A(1)

ENDIF

DADT(2)=KA*A(1)-K*A(2)-K23*A(2)+K32*A(3) -K24*A(2)+K42*A(4)

DADT(3)=K23*A(2)-K32*A(3)

DADT(4)=K24*A(2)-K42*A(4)

; For Cmax Tmax

TIMEATERDOSE=T-TIMEDOSE

CONCENTR = A(2)/V ; plasma concentration

IF (CONCENTR.GE.COM(1)) THEN

COM(1) = CONCENTR ; CMAX

COM(2) = TIMEATERDOSE ; TIME OF CMAX

ENDIF

DADT(5) = CONCENTR ; TO OBTAIN THE DAILY AUC, ;COMPARTMENTS 5 IS INTIALLY OFF AND OPENED AT EACH DOSING TIME THEN ;TURNED-OFF 24-H LATER (CMT DATAITEM)

$ERROR

IPRED = A(2)/V

IRES = DV-IPRED

PROP = IPRED*THETA(5)

ADD = THETA(6)

ADD_PORBX = THETA(12)

IF(DAT_TYPE.EQ.1) THEN ;SCALING SPARSE DATASET FROM PROBEX TO ACCOUNT FOR UNCERTAINITY IN DOSING TIME AND ADHERENCE

ADD = ADD_PORBX

ENDIF

W = SQRT(ADD**2+PROP**2)

IF (W.LE.0.000001) W=0.000001

IWRES = IRES/W

Y = IPRED + W*ERR(1)

;-------------------------------------------------------------------

IF(AMT>0) THEN

TIMEDOSE = TIME

AMOUNTDOSE = AMT

ENDIF

; For Cmax Tmax

C_MAX = COM(1) ; CMAX

T_MAX = COM(2) ; TIME OF CMAX

IF(AMT.GT.0) THEN

TIMEDOSE = TIME

AMOUNTDOSE = AMT

; Reset CMAX code when a new dose is given

COM(1)=0

COM(2)=0

ENDIF

AUC_24 = A(5)

AUC_INF=AMOUNTDOSE*BIO/CL

;To prevent simulation (ICALL==4) of negative values. It set a positive lower bound for Y, so that VPCs in the log-scale can be plotted

IF (ICALL==4.AND.Y<=0.00781) Y=0.003905

;--------------------------------------------------------------------------------

VARCL = BSVCL + BOVCL

VARBIO = BSVBIO + BOVBIO

VARAUC = BSVBIO + BOVBIO - BSVCL - BOVCL

AMOUNT_1 = A(1) ;drug amount at abs compartment

AMOUNT_2 = A(2) ;drug amount at central compartment

AMOUNT_3 = A(3) ;drug amount at PERIPH CMT

AMOUNT_4 = A(4) ;drug amount at PERIPH 2 CMT

;-----------------------------------------------------------------------

$ESTIMATION --------

1. World Health Organization (WHO). 2018. Technical report on critical concentrations for TB drug susceptibility testing of medicines used in the treatment of drug-resistant TBWho. Geneva, Switzerland.

2. Xu J, Wang B, Hu M, Huo F, Guo S, Jing W, Nuermberger E, Lu Y. 2017. Primary Clofazimine and Bedaquiline Resistance among Isolates from Patients with Multidrug-Resistant Tuberculosis. Antimicrob Agents Chemother 61.

3. Diacon AH, Dawson R, von Groote-Bidlingmaier F, Symons G, Venter A, Donald PR, van Niekerk C, Everitt D, Hutchings J, Burger DA, Schall R, Mendel CM. 2015. Bactericidal Activity of Pyrazinamide and Clofazimine Alone and in Combinations with Pretomanid and Bedaquiline. Am J Respir Crit Care Med 191:943–953.
